# The Association of Polycystic Ovary Syndrome and Gestational Hypertensive Disorders in a Diverse Community-Based Cohort

**DOI:** 10.1155/2019/9847057

**Published:** 2019-01-01

**Authors:** Diane Schneider, Joel R. Gonzalez, Miya Yamamoto, Jingrong Yang, Joan C. Lo

**Affiliations:** ^1^Department of Obstetrics and Gynecology, Kaiser Permanente Oakland Medical Center, USA; ^2^Division of Research, Kaiser Permanente Northern California, USA; ^3^Department of Obstetrics and Gynecology, Kaiser Permanente Hayward Medical Center, USA

## Abstract

**Purpose:**

To examine the association of polycystic ovary syndrome (PCOS) and pregnancy-induced hypertension (PIH) within a large population of pregnant women in an integrated healthcare system.

**Methods:**

This retrospective study utilized a source cohort of 1023 women with PCOS and 1023 women without PCOS who had a delivered pregnancy within Kaiser Permanente Northern California. Preexisting hypertension was defined by hypertension diagnosis, treatment, or elevated blood pressure prior to 20 weeks of gestation. The development of PIH, including gestational hypertension, preeclampsia/eclampsia, or HELLP (hemolysis, elevated liver enzymes, and low platelet count), was ascertained by chart review. Among women without preexisting hypertension who had a singleton pregnancy, the association of PCOS and PIH was examined using multivariable logistic regression.

**Results:**

Among 1902 women (910 PCOS) with singleton pregnancy, 101 (11.1%) PCOS and 36 (3.6%) non-PCOS women had preexisting hypertension and were excluded. Of the remaining 1765 women, those with PCOS (compared to non-PCOS) were slightly older (mean age 31.2 versus 30.7), more likely to be obese (39.6% versus 15.1%), nulliparous (63.8% versus 43.4%), and conceive with fertility treatment (54.1% versus 1.9%); they also had a higher incidence of PIH (10.8% versus 6.6%), including gestational hypertension (5.8% versus 3.6%) and preeclampsia or HELLP (4.9% versus 3.0%; all p<0.05). PCOS was associated with increased odds of PIH (odds ratio, OR 1.7, 95% confidence interval, CI 1.2-2.4), remaining significant after adjusting for age, race/ethnicity, nulliparity, and fertility treatment; however, findings were attenuated and no longer significant after adjusting for weight status (OR 1.1, CI 0.7-1.7). Maternal PCOS was also associated with preeclampsia/HELLP in unadjusted but not adjusted (OR 1.0, CI 0.5-1.9) analyses. Nulliparity and higher prepregnancy BMI were associated with PIH in both groups.

**Conclusion:**

Compared to women without PCOS, women with PCOS are at higher risk for PIH but this association was not independent of weight status.

## 1. Introduction

Polycystic ovary syndrome (PCOS) is a female endocrine disorder characterized by elevated androgen levels, ovulatory dysfunction, and polycystic ovarian morphology, as well as a constellation of classic clinical features that may include obesity, hirsutism, alopecia, acne, irregular menses, infertility, and high blood pressure [1–3]. Stein and Leventhal first described PCOS in 1935 when they reported on a series of seven female patients who presented with cystic ovaries, amenorrhea, and abnormal terminal hair growth [4]. Since that time, the diagnosis of PCOS among reproductive-aged women has become commonplace, with up to 10 percent of women presenting to gynecology clinic visits meeting criteria for diagnosis [5].

The diagnosis of PCOS has varied over the years and has included requirements of oligo/anovulation or polycystic ovaries, with androgen excess [6, 7]. However, recent consensus and current international guidelines affirm the European Society for Human Reproduction and Embryology (ESHRE)/American Society for Reproductive Medicine (ASRM) Rotterdam criteria of androgen excess, oligoovulation or anovulation, and polycystic ovaries on ultrasound (two of three requirements met) [1, 8] as the most inclusive diagnostic criteria for PCOS and the most commonly used internationally [9, 10].

There is evidence that women with PCOS may be at greater risk for various obstetric complications, including gestational hypertension and preeclampsia [11, 12], gestational diabetes mellitus (GDM), premature birth, and cesarean delivery [13]. Gestational hypertensive disorders are the leading causes of maternal morbidity and mortality, affecting 3-10% of all pregnancies including a subset of pregnancies resulting in preeclampsia [14, 15], and contribute up to 16% of maternal deaths in developed nations [16]. Two large meta-analyses found a two- to four-fold increased rate of pregnancy-induced hypertension (PIH) as well as preeclampsia in women with PCOS [13, 17], and a large nationwide study conducted in Sweden found that PCOS was associated with a 1.5-fold increased odds of preeclampsia, even after adjusting for body mass index (BMI), parity, and use of reproductive technology [18].

The primary objective of this study was to investigate hypertension in pregnancy and the incidence of gestational hypertension and preeclampsia in a racially and ethnically diverse cohort of pregnant women with and without PCOS and examine the independent association of maternal PCOS and gestational hypertensive disorders among women without preexisting hypertension. A secondary aim was to examine the clinical and demographic predictors of gestational hypertensive disorders among the subset of women with PCOS.

## 2. Materials and Methods

This study was conducted using data from Kaiser Permanente Northern California (KPNC), a large healthcare system that had more than three million members and over 30,000 live births per year during 2002-2005. We used a previously identified cohort of 1023 pregnant women with PCOS and 1023 pregnant women without PCOS aged 16-44 years old and matched by delivery year during 2002-2005 [19], focusing on the subset of 1902 women with singleton pregnancy [19, 20]. Women with PCOS were identified based on clinical diagnosis that was validated by chart review, using the ESHRE/ASRM Rotterdam criteria for PCOS as previously described [1]. Prepregnancy body mass index (BMI) and preexisting hypertension were examined by chart review, with preexisting hypertension defined by prepregnancy diagnosis or treatment of hypertension or evidence of elevated blood pressure defined by systolic blood pressure (SBP) ≥140 mmHg or diastolic blood pressure (DBP) ≥ 90 mmHg on at least two separate clinic visits prior to 20 weeks of gestation. Gestational hypertensive disorders, including preeclampsia and other subtypes, were confirmed and adjudicated by detailed obstetrics/gynecology physician chart review using working definitions based on guidelines from The American Congress of Obstetricians and Gynecologists [21, 22] during the period of our study. Patients were classified as having gestational hypertension, preeclampsia, preeclampsia with severe features, or HELLP syndrome. The following definitions were used: gestational hypertension (SBP ≥140 mmHg or DBP ≥90 mmHg occurring after 20 weeks of gestation in a woman with previously normal blood pressure without proteinuria), preeclampsia (hypertension with new onset proteinuria >300 mg over 24-hour collection or urine protein 1+ or 30mg on dipstick or 100mg of protein on formal urinalysis), preeclampsia with severe features (meeting diagnostic criteria for preeclampsia plus any one or more of the following: severe range blood pressures as defined as SBP ≥160 mmHg or DBP ≥110 mmHg on at least two occasions, ≥5gm of protein on 24 hour total urine protein collection, 3+ or 4+ on urine dip on two occasions, ≥ 300 mg on urinalysis on at least two occasions, or symptoms including clinically significant headache, visual disturbances, pulmonary edema, epigastric or right upper quadrant pain, intrauterine growth restriction, or oliguria), HELLP syndrome (hemolysis, clinically significant elevated liver enzymes and low platelet count), super-imposed preeclampsia (new onset proteinuria or significant worsening of kidney function on preexisting hypertension), and eclampsia (new onset seizures in a woman with preeclampsia). For these analyses, women were classified as having chronic hypertension, gestational hypertension, preeclampsia/eclampsia, or HELLP.

### 2.1. Identification of Baseline Characteristics

Maternal age, race/ethnicity, parity, reproductive history, method of conception, use of fertility medication (clomiphene citrate, gonadotropins, and metformin) and assisted reproduction, prepregnancy BMI, and evidence of prior hypertension diagnoses or treatment with blood pressure-lowering medication were examined by individual chart review. In addition, prenatal visit records were examined for evidence of elevated systolic (≥140 mmHg) and/or diastolic (≥90 mmHg) blood pressure on at least two occasions prior to 20 weeks of gestation to identify additional women with preexisting hypertension without prior hypertension diagnosis or treatment.

### 2.2. Statistical Analyses

Differences between subgroups were examined by Student's t-test or Wilcoxon rank sum test for continuous variables and the chi-squared test for categorical variables. Multivariable logistic regression was used to examine the independent relationship of PCOS status and other relevant clinical factors with PIH (gestational hypertension, preeclampsia/eclampsia, or HELLP). These included age, race/ethnicity, nulliparity, fertility treatment (fertility medication or* in vitro* fertilization), and weight status (BMI). Among women with PCOS, we also examined clinical predictors of PIH. All analyses were conducted using STATA version 10.1 (StataCorp LP, College Station, TX, USA). A two-sided p-value of 0.05 was chosen as the criterion for statistical significance.

## 3. Results

Among the 1902 women (910 with PCOS) with a delivered singleton pregnancy, 101 (11.1%) PCOS and 36 (3.6%) non-PCOS women were classified as having chronic hypertension, based on hypertension diagnosis, antihypertensive treatment, or evidence of elevated blood pressure prior to 20 weeks of gestation from clinical records in the absence of known hypertension and/or treatment (51 PCOS and 17 non-PCOS women in this latter group). These 137 women were excluded from further analyses in this study which focused on examining the risk of PIH in women without evidence of chronic hypertension.

Among the remaining 1765 women without preexisting hypertension (809 PCOS, 956 non-PCOS), the average age was 31.0 ± 5.4 years, slightly higher among women with PCOS (31.2 ± 4.4) compared to those without PCOS (30.7 ± 6.0, p=0.04; Table 1). As previously reported [19], women with PCOS were more likely to be obese (39.6% versus 15.1%), more likely to be nulliparous (63.8% versus 43.4%), and to receive fertility treatment for successful conception (54.1% versus 1.9%) compared to women without PCOS (p<0.01). The cohort also manifested substantial ethnic diversity, which varied slightly by PCOS status (40.3% white, 3.6% black, 27.0% Hispanic, and 26.0% Asian versus 40.3% white, 6.8% black, 24.4% Hispanic, and 24.9% Asian for non-PCOS, p=0.04).

A total of 8.5% developed PIH, including gestational hypertension (4.6%), preeclampsia (3.6%) and HELLP (0.3%), although none developed eclampsia. Comparing women with and without PCOS, 5.8% and 3.6% (p=0.02) developed gestational hypertension, and 4.9% and 3.0% (p=0.04) developed preeclampsia or HELLP syndrome, respectively. The overall incidence of PIH in women with PCOS was 10.8% compared to 6.6% in women without PCOS (p<0.01).

The clinical characteristics by PCOS and PIH status are shown in Table 2. Within each clinical subgroup, no statistically significant differences in age, race/ethnicity and overall gravidity were noted, although nulliparity and prepregnancy BMI differed by PIH status. Using multivariable logistic regression analyses, PCOS was associated with increased odds of PIH (odds ratio 1.7, 95% confidence interval, CI 1.2-2.4) in unadjusted analyses and after adjusting for differences in age, race/ethnicity, nulliparity and fertility treatment (adjusted OR 1.5, CI 1.0-2.4). However, this association was attenuated and no longer significant after adjusting for prepregnancy BMI (adjusted OR 1.1, CI 0.7-1.7). When examining the association of PCOS and preeclampsia or HELLP specifically, PCOS status was associated with preeclampsia/HELLP in unadjusted analyses (OR 1.7, CI 1.0-2.7) but no association was evident in adjusted analyses (adjusted OR 1.0, CI 0.5-1.9).

Among the 809 women with PCOS, adjusting for differences in age, race/ethnicity, nulliparity, fertility treatment and prepregnancy BMI, both nulliparity (adjusted OR 2.3, CI 1.3-3.9) and higher BMI (adjusted OR 3.3, CI 1.5-7.1 for BMI 25-29; OR 5.8, CI 2.7-12.4 for BMI 30-39; and OR 4.2, CI 1.6-10.9 for BMI ≥40 compared to BMI <25 kg/m^2^) were significant predictors of development of PIH. Similar associations of nulliparity and PIH (adjusted OR 2.3, CI 1.3-4.0) were seen in non-PCOS women, whereas for weight status (BMI), only class III obesity (BMI ≥40 kg/m^2^) was associated with higher risk of PIH compared to normal weight non-PCOS women (adjusted OR 17, CI 5.1-54) but the number of non-PCOS women in this BMI category was extremely small (1.5%).

## 4. Discussion

This is one of the first studies to assess the relationship of maternal PCOS and the development of PIH in an ethnically diverse community-based cohort of women with singleton pregnancy in the western US. As PCOS continues to become increasingly recognized in the clinical setting, with larger numbers of women with PCOS achieving successful pregnancy, there is a need to better understand pregnancy outcomes within this population and to examine findings within integrated health systems.

There is a growing body of evidence suggesting that PCOS is associated with a greater risk of adverse outcomes in pregnancy. These include hypertensive disease/preeclampsia, gestational diabetes, preterm delivery, low birth weight, neonatal intensive care unit admission, and increased likelihood of cesarean delivery [12, 13, 17–19, 23]. Population data and aggregate meta-analyses conducted across multiple studies examining adverse outcomes in PCOS pregnancies all show an increased risk for PIH and preeclampsia in women with PCOS, with a preponderance of data from Scandinavia [13, 17, 24]. Our analyses contribute to these findings by reporting data from a single integrated health system serving a diverse Northern California population, where women with preexisting hypertension were excluded from the analyses. Although we did not find an independent association between PIH and maternal PCOS when considering differences in underlying weight status, we did identify clinical predictors of PIH that may inform clinical care of women with PCOS who become pregnant, including nulliparity and higher BMI.

While our study presents data from a large and diverse real-world population of women with PCOS achieving sustained pregnancy, there are some important limitations to consider. First, the criteria for diagnosing preeclampsia has since been updated by the American College of Obstetricians and Gynecologist Task Force on Hypertension in Pregnancy in 2013 [25], whereas the diagnosis of preeclampsia in this study accurately reflects the typical clinical practice of obstetricians functioning in the acute setting of labor and delivery units during our study period. These criteria were consistent for both PCOS and non-PCOS women and thus do not affect the relative comparison between groups. Second, the size of this study and relatively small number of PIH and preeclampsia events limit the ability to examine small differences by clinical status, including PIH severity. Third, we did not assess other factors that might have contributed to PIH risk, including weight gain, hormone levels and other cardiovascular or metabolic biomarkers.

In light of the growing body of literature examining potentially adverse pregnancy outcomes in women with PCOS, we identified several possible areas for future study. Notably, PCOS presents along a spectrum of symptoms and clinical severity [3, 26]. In assessing PCOS, some endocrinologists consider the degree of hyperandrogenism, including subjective clinical presentation, and objective serum testosterone levels, to determine the severity of disease. Several studies have investigated the degree to which a patient's androgen status affects pregnancy outcome. To date, the data appear inconclusive, with Naver et al. [27] suggesting a correlation between hyperandrogenemia status and increased rates of negative obstetric outcomes and Mumm et al. [28] finding no difference in outcomes among PCOS women by androgen status or PCOS phenotype. It has also been suggested that the degree to which a woman manifests hyperandrogenism, obesity, and related health conditions, including insulin resistance, affects the risk for PIH [13, 29]. Indeed, we found that weight status appeared to mediate in part the observed relationship between PCOS and the development of PIH. Future studies in larger population cohorts might endeavor to investigate gestational outcomes by severity of PCOS to determine whether specific risk classification might be relevant for the individual patient. The impact of preconception weight control in PCOS women with obesity, a modifiable risk factor, may also be an important consideration.

Our study found that maternal PCOS status was associated with increased risk of developing gestational hypertensive disease, but this association was not independent of pregestational weight status. This study supports an increasing number of published reports demonstrating that women with PCOS have a higher burden of high-risk pregnancies. Whether the underlying etiology results from inherent factors of PCOS or other related characteristics that are part of the PCOS phenotype (e.g., obesity, insulin resistance, and hyperandrogenism) are not known, providers should be aware that this clinical population is more likely to develop adverse pregnancy outcomes as compared to their non-PCOS peers. A final important lesson for clinicians caring for pregnant women with PCOS is the high rate of chronic hypertension (11.1%) when compared to non-PCOS women (3.6%). While we excluded these cases from our analyses, we noted that many women were classified as having preexisting hypertension based on high blood pressure identified during early prenatal visits (prior to 20 weeks of gestation). As a common point of entry into the healthcare system, pregnancy provides women the opportunity to address chronic health issues that might otherwise be missed. In this way, pregnancy also offers health care providers a potential period of intervention to potentially reduce end-organ damage associated with untreated disease, which is particularly relevant for women with PCOS who have other metabolic risk factors.

## Figures and Tables

**Table 1:** Baseline characteristics of women with singleton pregnancy and no preexisting hypertension by polycystic ovary syndrome status.

	PCOS Women	Non-PCOS Women
	N = 809	N = 956
Age (mean ± SD)	31.2 ± 4.4^*∗*^	30.7 ± 6.0

Race/Ethnicity	*∗*	
White	326 (40.3%)	385 (40.3%)
Black	29 (3.6%)	65 (6.8%)
Hispanic	218 (27.0%)	233 (24.4%)
Asian	210 (26.0%)	238 (24.9%)
Other	26 (3.2%)	35 (3.7%)

Nulliparous	516 (63.8%)^*∗∗*^	415 (43.4%)

Fertility Treatment^†^	438 (54.1%)^*∗∗*^	18 (1.9%)

Pre-pregnancy BMI ≥30.0 kg/m^2^)	320 (39.6%)^*∗∗*^	144 (15.1%)

^*∗*^p<0.05 and ^*∗∗*^p<0.001 comparing PCOS versus non-PCOS using the t-test for continuous variables and the chi-squared test for categorical variables.

^†^Fertility treatment includes receipt of fertility medications and assisted reproductive technology (e.g., *in vitro* fertilization).

**Table 2:** Clinical characteristics of women without preexisting hypertension by polycystic ovary syndrome (PCOS) and pregnancy-induced hypertension (PIH) status.

	**No PCOS**			**PCOS**	
	No PIH	PIH		No PIH	PIH
	(n=893)	(n =63)		(n=722)	(n= 87)
Maternal age (mean ± SD)	30.8 ± 6.0	29.7 ± 6.2		31.2 ± 4.4	31.5 ± 4.8
Maternal age ≥ 35 years, n (%)	230 (25.8%)	16 (25.4%)		144 (19.9%)	20 (23.0%)
Race/Ethnicity, n (%)					
White	355 (39.8%)	30 (47.6%)		285 (39.5%)	41 (47.1%)
Black	58 (6.5%)	7 (11.1%)		26 (3.6%)	3 (3.5%)
Hispanic	220 (24.6%)	13 (20.6%)		197 (27.3%)	21 (24.1%)
Asian	227 (25.4%)	11 (17.5%)		192 (26.6%)	18 (20.7%)
Other	33 (3.7%)	2 (3.2%)		22 (3.1%)	4 (4.6%)
Gravidity (median, IQR)	2 (1-3)	2 (1-3)		2 (1-3)	2 (1-2)
Nulliparity, n (%)	377 (42.2%)	38 (60.3%)^*∗*^		451 (62.5%)	65 (74.7%)^*∗*^
Pre-pregnancy BMI, n (%)		*∗*			*∗*
<25 kg/m^2^	538 (60.3%)	30 (47.6%)		228 (31.6%)	9 (10.3%)
25-29 kg/m^2^	225 (25.2%)	18 (28.6%)		225 (31.2%)	27 (31.0%)
30-39 kg/m^2^	121 (13.6%)	9 (14.3%)		204 (28.3%)	40 (46.0%)
≥40 kg/m^2^	8 (0.9%)	6 (9.5%)		65 (9.0%)	11 (12.6%)

Column percentages are represented.

^*∗*^p < 0.05 comparing no PIH and PIH using the t-test for continuous variables and the chi-squared test for categorical variables.

## Data Availability

The summary data tables used to support the findings of this study are available from the corresponding author upon request. Individual level data are not available for external request.

## References

[1] Rotterdam ESHRE/ASRM-Sponsored PCOS Consensus Workshop Group (2004). Revised 2003 consensus on diagnostic criteria and long-term health risks related to polycystic ovary syndrome. *Human Reproduction*.

[2] Legro R. S., Arslanian S. A., Ehrmann D. A. (2013). Diagnosis and treatment of polycystic ovary syndrome: an endocrine society clinical practice guideline. *The Journal of Clinical Endocrinology & Metabolism*.

[3] (2018). ACOG Practice Bulletin No. 194. *Obstetrics & Gynecology*.

[4] Stein I. F., Leventhal M. L. (1935). Amenorrhea associated with bilateral polycystic ovaries. *American Journal of Obstetrics & Gynecology*.

[5] Cahill DJ., O'Brien K. Polycystic ovary syndrome (PCOS): metformin. *BMJ Clinical Evidence*.

[6] Azziz R., Carmina E., Dewailly D. (2006). Positions statement: criteria for defining polycystic ovary syndrome as a predominantly hyperandrogenic syndrome: an Androgen Excess Society guideline. *The Journal of Clinical Endocrinology & Metabolism*.

[7] Zawadski J. K., Dunaif A., Dunaif A., Givens J. R., Haseltine F. P., Merriam G. R. (1992). Diagnostic criteria for polycystic ovary syndrome: Towards a rational approach. *Polycystic Ovary Syndrome*.

[8] Sirmans S. M., Pate K. A. (2013). Epidemiology, diagnosis, and management of polycystic ovary syndrome. *Clinical Epidemiology*.

[9] Mortada R., Williams T. (2015). Metabolic Syndrome: Polycystic Ovary Syndrome. *FP Essentials*.

[10] International evidence-based guideline for the assessment and management of polycystic ovary syndrome. https://www.monash.edu/__data/assets/pdf_file/0004/1412644/PCOS-Evidence-Based-Guideline.pdf.

[11] De Vries M. J., Dekker G. A., Schoemaker J. (1998). Higher risk of preeclampsia in the polycystic ovary syndrome. A case control study. *European Journal of Obstetrics & Gynecology and Reproductive Biology*.

[12] Palomba S., de Wilde M. A., Falbo A., Koster M. P., La Sala G. B., Fauser B. C. (2015). Pregnancy complications in women with polycystic ovary syndrome. *Human Reproduction Update*.

[13] Qin J. Z., Pang L. H., Li M. J., Fan X. J., Huang R. D., Chen H. Y. (2013). Obstetric complications in women with polycystic ovary syndrome: a systematic review and meta-analysis. *Reproductive Biology and Endocrinology*.

[14] Duley L. (2009). The global impact of pre-eclampsia and eclampsia. *Seminars in Perinatology*.

[15] Wallis A. B., Saftlas A. F., Hsia J., Atrash H. K. (2008). Secular trends in the rates of preeclampsia, eclampsia, and gestational hypertension, United States, 1987–2004. *American Journal of Hypertension*.

[16] Khan K. S., Wojdyla D., Say L., Gülmezoglu A. M., van Look P. F. (2006). WHO analysis of causes of maternal death: a systematic review. *The Lancet*.

[17] Boomsma C. M., Eijkemans M. J. C., Hughes E. G., Visser G. H. A., Fauser B. C. J. M., Macklon N. S. (2006). A meta-analysis of pregnancy outcomes in women with polycystic ovary syndrome. *Human Reproduction Update*.

[18] Roos N., Kieler H., Sahlin L., Ekman-Ordeberg G., Falconer H., Stephansson O. (2011). Risk of adverse pregnancy outcomes in women with polycystic ovary syndrome: Population based cohort study. *British Medical Journal*.

[19] Yamamoto M., Feigenbaum S. L., Crites Y. (2012). Risk of preterm delivery in non-diabetic women with polycystic ovarian syndrome. *Journal of Perinatology*.

[20] Feigenbaum S. L., Crites Y., Hararah M. K., Yamamoto M. P., Yang J., Lo J. C. (2012). Prevalence of cervical insufficiency in polycystic ovarian syndrome. *Human Reproduction*.

[21] ACOG Committee on Practice Bulletins (2001). ACOG Practice Bulletin. Chronic hypertension in pregnancy. *Obstetrics & Gynecology*.

[22] (2002). ACOG Practice Bulletin No. 33: Diagnosis and Management of Preeclampsia and Eclampsia. *Obstetrics & Gynecology*.

[23] Lo J. C., Feigenbaum S. L., Escobar G. J., Yang J., Crites Y. M., Ferrara A. (2006). Increased prevalence of gestational diabetes mellitus among women with diagnosed polycystic ovary syndrome: A population-based study. *Diabetes Care*.

[24] Kjerulff L. E., Sanchez-Ramos L., Duffy D. (2011). Pregnancy outcomes in women with polycystic ovary syndrome: a metaanalysis. *American Journal of Obstetrics & Gynecology*.

[25] American College of Obstetricians and Gynecologists (2013). Hypertension in pregnancy. Report of the American College of Obstetricians and Gynecologists' Task Force on Hypertension in Pregnancy.. *Obstetrics & Gynecology*.

[26] Palomba S., Santagni S., Falbo A., La Sala G. B. (2015). Complications and challenges associated with polycystic ovary syndrome: current perspectives. *International Journal of Women's Health*.

[27] Naver K. V., Grinsted J., Larsen S. O. (2014). Increased risk of preterm delivery and pre-eclampsia in women with polycystic ovary syndrome and hyperandrogenaemia. *BJOG: An International Journal of Obstetrics & Gynaecology*.

[28] Mumm H., Jensen D. M., Sørensen J. A. (2015). Hyperandrogenism and phenotypes of polycystic ovary syndrome are not associated with differences in obstetric outcomes. *Acta Obstetricia et Gynecologica Scandinavica*.

[29] Palomba S., Falbo A., Russo T., Tolino A., Orio F., Zullo F. (2010). Pregnancy in women with polycystic ovary syndrome: The effect of different phenotypes and features on obstetric and neonatal outcomes. *Fertility and Sterility*.

